# Projektion der COVID-19-Epidemie in Deutschland

**DOI:** 10.1007/s10273-020-2631-5

**Published:** 2020-04-22

**Authors:** Jean Roch Donsimoni, René Glawion, Bodo Plachter, Klaus Wälde

**Affiliations:** 1 FB Rechts- und Wirtschaftswissenschaften, Johannes Gutenberg-Universität Mainz, Jakob Welder Weg 4, 55128 Mainz, Deutschland; 2 Fakultät für Wirtschafts- und Sozialwissenschaften, Universität Hamburg, Von-Melle-Park 5, 20148 Hamburg, Deutschland; 3 Institut für Virologie, Johannes Gutenberg-Universität Mainz, Obere Zahlbacher Straße 67, 55131 Mainz, Deutschland; 4 FB Rechts- und Wirtschaftswissenschaften, Johannes Gutenberg-Universität Mainz, Jakob Welder Weg 4, 55128 Mainz, Deutschland

## Abstract

Die COVID-19-Epidemie wird in Deutschland im optimistischen Szenario mindestens bis Juli 2020 dauern, im normalen Szenario bis August. Öffentliche Maßnahmen wie Kontaktverbote flachen den Anstieg der Zahl der Erkrankungen temporär ab und verlängern die Dauer der Epidemie. In der Spitze sind im optimistischen Szenario gleichzeitig bis zu 200.000 Menschen erkrankt, im normalen Szenario liegen die Werte um 1 Million. Langfristig werden im normalen Szenario 6 % der Bevölkerung (gemeldet) erkrankt gewesen sein.

## Abstract (en)

The authors model the evolution of the number of confi rmed cases of COVID-19 in Germany. Their theoretical framework builds on a continuous time Markov chain with four physical states: healthy, sick, recovered or asymptomatic infected, and dead. Their quantitative solution matches the number of sick individuals based on the most recent fi gures with the share of sick individuals following from infection rates and sickness probabilities. They employ this framework to study the expected peak of the number of sick individuals in a scenario without public regulation of social contacts. They also study the impact of public regulations. For all scenarios, they report the expected end of the COVID-19 epidemic.

Die durch den Coronavirus SARS-CoV-2 ausgelöste Pandemie hält Deutschland und die Welt fest im Griff. Deren Bedeutung für die gesamtgesellschaftliche Gesundheit und Wirtschaft und für das allgemeine mentale Wohlempfinden in Deutschland benötigt keiner Betonung. Die dringendste Frage dieser Zeit scheint zu sein: Wie lange geht das noch? Wir verwenden alles uns zugängliche Wissen aus der Virologie, Epidemiologie und den Methoden der Volkswirtschaftslehre bzw. der Stochastik, um darauf eine Antwort zu geben. Neben dem Aspekt der Dauer stellen sich noch die folgenden Fragen: Wie groß ist die Zahl der erkrankten Personen im Zeitverlauf, die wir zu erwarten haben? Wie hoch wird die Zahl der erkrankten Personen sein, die sich im Zeitverlauf mit dem Coronavirus je infizieren? Auf der letzten Frage liegt das Hauptaugenmerk der breiten Öffentlichkeit, der Politik und der Krankenhäuser, um die Bereitstellung von Krankenhausbetten planen zu können. Nicht zuletzt stellt sich die Frage, welche Auswirkungen aktuelle Politikmaßnahmen auf den Verlauf der Epidemie haben.

**René Glawion**, M.Sc., ist wissenschaftlicher Mitarbeiter an der Fakultät für Wirtschafts- und Sozialwissenschaften der Universität Hamburg.

**Prof. Dr. Bodo Plachter** ist stellvertretender Direktor am Institut für Virologie der Johannes Gutenberg-Universität Mainz.

**Prof. Dr. Klaus Wälde** ist Professor für Makroökonomik am Fachbereich Rechts- und Wirtschaftswissenschaften der Johannes Gutenberg-Universität Mainz und Mitglied in den Forschungsnetzwerken CESifo und IZA.

## Coronavirus: ein Blick auf andere Länder

Als Hintergrund zur Beurteilung der Lage in Deutschland betrachten wir zunächst die Verläufe der Epidemie in der Provinz Hubei in China, in Südkorea und Japan. Diese Entwicklungen werden eine zentrale Rolle in der Einschätzung des weiteren COVID-19-Verlaufs in Deutschland spielen.

Abbildung 1 zeigt die Zahl der als erkrankt gemeldeten Personen in diesen drei Regionen bzw. Ländern. Wir se-

* Dies ist eine Zusammenfassung von „Projecting the Spread of COVID19 for Germany" geschrieben von den gleichen Autoren (Donsimoni et al., 2020). Die Arbeit war in der Schnelle der Zeit nur durch wertvolle Hinweise vieler Kollegen möglich. Wir danken Matthias Birkner, Christian Dormann, Oliver Emrich, Hoang Khieu, Dietmar Leisen, Kai Pastor und Olaf Posch. Wir danken vor allem unseren Kollegen aus dem medizinischen und vor allem epidemiologischen Bereich für wertvolle Kommentare. Dies sind unter anderem Matthias an der Heiden, Michael Bauer, Julien Riou, Perikles Simon, Martin Wolkewitz und Mitglieder der Modellierungsgruppe der Deutschen Gesellschaft für Epidemiologie (DGEpi). Eine Aktualisierung der Vorhersagen erfolgt auf https://www.macro.economics.uni-mainz.de/corona-blog/ (2. April 2020).

Abbildung 1

**Verlauf der Fallzahlen in Hubei, Südkorea und Japan, 2020**

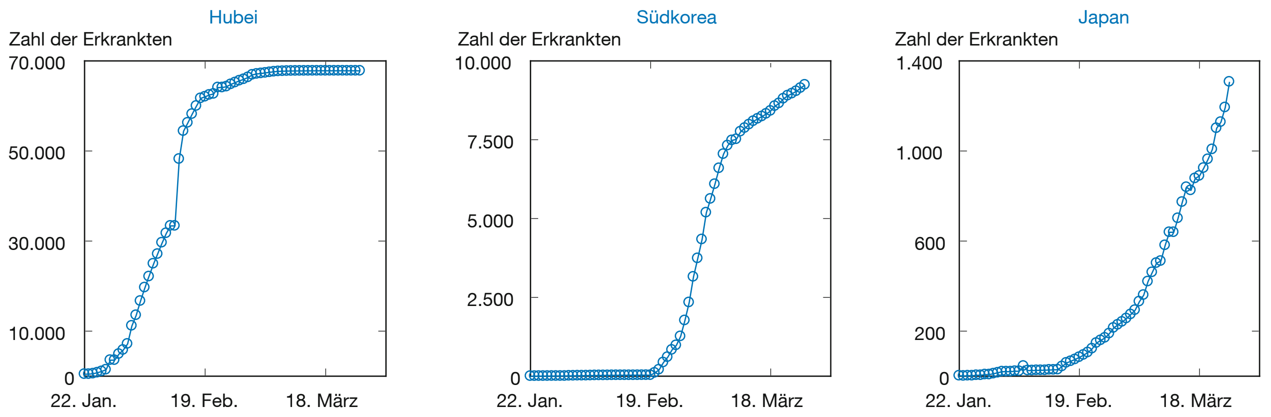

Quelle: Donsimoni, J. R., R. Glawion, B. Plachter und K. Wälde (2020), Projecting the Spread of COVID19 for Germany, https://www.macro.economics.uni-mainz.de/files/2020/03/Donsimoni-Glawion-Plachter-Wälde-2020-Projecting-spread-of-CoV-2-in-Germany-1.pdf (30. März 2020); nach Daten der Johns Hopkins University (USA).

hen, und das scheint uns etwas Hoffnung schöpfen zu lassen, dass die Zahl der Infizierten in Hubei bereits gegen eine Konstante zu konvergieren scheint. Diese liegt bei etwa 1 von 1.000 Personen. Wenn also die Epidemie in Hubei tatsächlich vorüber ist und die Daten ausreichend hohe Qualität haben, dann ist am Ende der COVID-19-Epidemie in Hubei 1 von 1.000 Personen erkrankt (oder, in den meisten Fällen, erkrankt gewesen). Anders ausgedrückt sind 999 von 1.000 Personen entweder die ganze Zeit gesund gewesen, oder auch infiziert, aber mit einem so milden Verlauf der Symptome, dass sie eben nicht als erkrankt erfasst wurden.

Ein ähnlicher Verlauf ist in Südkorea zu erahnen, wo sich die Zahl der gemeldeten Erkrankten ebenfalls gegen eine obere Schranke zu entwickeln scheint. Diese liegt bei etwa 3 von 5.000 erkrankten Personen. Die Datenqualität in Südkorea wird üblicherweise höher eingeschätzt als die aus Hubei. Es wird jedoch häufig betont, dass Südkorea sich vermutlich nicht am Ende der COVID-19-Epidemie befindet. Vielmehr ist es gelungen, die Ausbreitung zu stoppen, was jedoch im Umkehrschluss bedeutet, dass es eine Vielzahl von nicht-immunen Einwohnern Südkoreas gibt. Am Ende einer typischen Epidemie sind etwa 50 % bis 60 % einer Bevölkerung infiziert bzw. haben Antikörper gebildet, sind also immun.[1] Wenn eine Epidemie unterdrückt wird, dann ist diese Immunität in der Bevölkerung nicht ausreichend vorhanden. Es könnte also jederzeit zu einem erneuten Ausbruch der Epidemie kommen.

1 Dies ergibt sich aus dem Ausdruck (R0-1)/R0, wobei R0 die Basisreproduktionszahl ist. Sie gibt an, wie viele Personen durchschnittlich von einer infizierten Person angesteckt werden. Diese Basisreproduktionszahl liegt für eine typische Grippe bei 2 (Mikolajczyk et al., 2009) und wird für CoV-2 in Wuhan auf ca. 2 bis 2,5 geschätzt (Riou und Althaus, 2020).

In Japan ist dagegen noch kein solcher Verlauf bzw. noch kein Abflachen der Kurve zu erkennen. Leider ist Japan der typische Fall für alle anderen Länder, für die ausreichend Datenmaterial zur Verfügung steht. Diese Länder befinden sich alle eher am Anfang oder in der Mitte des Epidemieverlaufs.

## Vorgehensweise bei der Projektion des Verlaufs der COVID-19-Epidemie in Deutschland

Unser Modell teilt die Bevölkerung in Deutschland in vier Gruppen auf: (1) gesund ohne Infektion, (2) an COVID-19 erkrankt, (3) gesund nach Genesung oder nach einer (annähernd) symptomfreien Infektion, und (4) verstorben. Wir beschreiben die Entwicklung der Zahl der Personen in diesen Gruppen ab 24. Februar 2020 (der erste Tag mit einer Meldung von Erkrankten in unserer Datenquelle) bis Ende des Jahres. Unter Verwendung entsprechender statistischer Methoden können wir mit unseren Modellgruppen die Entwicklung der Erkrankten in den Daten exakt nachzeichnen. Unser Modell beschreibt also die Entwicklung vom 24. Februar bis zum aktuellen Rand (fast vollkommen) exakt. Als Datengrundlage dienen uns die Meldungen des Robert Koch-Instituts (RKI, 2020).

Mithilfe des Modells und der vorliegenden Beobachtungen des RKI können wir die Entwicklung in Deutschland für die nächsten zwei bis drei Wochen gut vorhersagen. Die viel wichtigeren Fragen bezüglich des weiteren Verlaufs, einer maximalen Belastung des Gesundheitssystems und eines Endes der Epidemie können wir aufgrund dieser Datengrundlage jedoch nicht beantworten. Warum? Unser Modell (eine Markovkette in kontinuierlicher Zeit mit vier Zuständen und zustandsabhängigen Übergangsraten) sagt uns, dass wir dazu vor allem zwei weitere Daten benötigen: Wie hoch ist der Anteil der mit CoV-2 infizierten Individuen, die auch tatsächlich erkranken? Es ist allgemein bekannt, dass nicht alle Infektionen zu einer Erkrankung führen. Es wird sogar davon ausgegangen, dass üblicherweise die wenigstens Infektionen zu Erkrankungen führen. Es wird vermutet, dass eine Infektion mit Coronaviren (CoV-2) bei nur etwa 20 % zu Symptomen führen. Diese Zahl ist jedoch mit großer Unsicherheit verbunden. Die zweite Parameterinformation, die wir benötigen, ist der Anteil der am Ende der COVID-19-Epidemie infizierten Personen. Diese Zahl scheint mit noch viel größerer Unsicherheit behaftet zu sein. Es wird üblicherweise davon ausgegangen, dass diese bei etwa 50 % bis 60 % liegt. Wenn 60 % langfristig infiziert sind und 20 % der Infizierten erkranken, dann kommen wir auf 12 % Erkrankte nach Ende der Epidemie. Wenn wir davon ausgehen, dass von den Erkrankten nur etwa die Hälfte gemeldet wird (die andere Hälfte geht nicht zum Arzt oder die Symptome werden als „normale Erkältung“ eingestuft, oder es wird aus anderen Gründen kein SARS-CoV-2-Test durchgeführt), dann liegen am Ende der Epidemie 6 % gemeldete Erkrankte für Deutschland vor. Das wären bei 83,1 Mio. Einwohnern knapp 5 Mio. Erkrankte.

Wenn wir diese langfristigen Zusammenhänge ebenfalls in die Berechnung durch unser Modell einfließen lassen, dann können wir den Verlauf der Epidemie ziemlich gut vorhersagen. Aber natürlich ist die Vorhersage nur so gut, wie die darin enthaltenen Annahmen, also vor allem die zwei zentralen Parameter „Erkrankungswahrscheinlichkeit“ und „langfristige Infektionsrate“. Da diese Parameter mit großer Unsicherheit verbunden sind (es gab noch keine COVID-19-Epidemie, in der die Medizin diese Werte ausreichend sicher hätte erheben können), berücksichtigen wir in unserer Projektion auch noch ein zweites Szenario, das „optimistische Hubei-Szenario“.

In Hubei scheint der langfristige Anteil der gemeldet Erkrankten in der Gesamtbevölkerung bei ca. 1 von 1.000 zu liegen. Um etwas vorsichtiger zu sein und um die Zahlen besser mit den bisherigen Kenngrößen zu vergleichen heben wir diesen Wert auf 6 von 1.000 an. Das mag eine manchmal vermutete unvollständige Datenerhebung korrigieren. Mit dieser Korrektur ist mit 6 von 1.000 der langfristige Wert in Hubei um einen Faktor 10 niedriger

Abbildung 2

**Zahl der Erkrankten über den Verlauf der COVID-19-Epidemie in Deutschland, 2020**

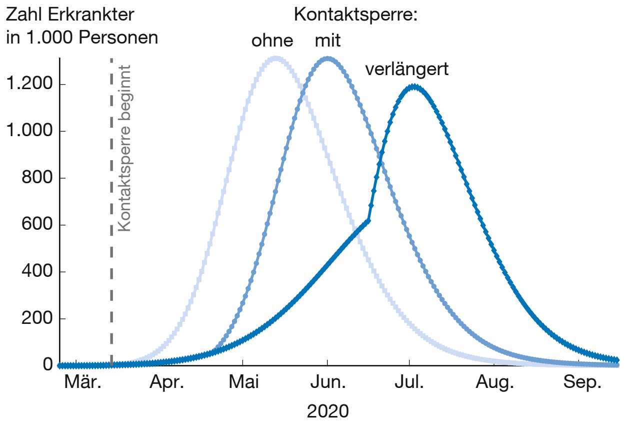

Anmerkung: Öffentliche Eingriffe werden in der Abbildung kurz ‚Kontaktsperre‘ genannt.

Quelle: Donsimoni, J. R., R. Glawion, B. Plachter und K. Wälde (2020), Projecting the Spread of COVID19 for Germany, https://www.macro.economics.uni-mainz.de/files/2020/03/Donsimoni-Glawion-Plachter-Wälde-2020-Projecting-spread-of-CoV-2-in-Germany-1.pdf (30. März 2020); nach Daten der Johns Hopkins University (USA).

als der Wert von 6 von 100, also 6 % für Deutschland, der sich aus den üblichen Parametern wie oben beschrieben ergibt. Wir haben also ein optimistisches Hubei-Szenario und ein normales Szenario.

## Eine ungedämpfte Epidemie und der Effekt temporärer Gesundheitsmaßnahmen

Abbildung 2 zeigt den Verlauf der Epidemie ab 24. Februar bis Ende September 2020. Sie zeigt die Zahl der an COVID-19 erkrankten Patienten im Zeitverlauf. Es ist wichtig zu betonen, dass dies nicht den Zahlen entspricht, die vom RKI gemeldet werden. Das RKI meldet die Zahl der je an COVID-19 erkrankten Individuen. In unserem Modell ist es ein Einfaches, diese Zahl um die Genesungen (wir nehmen eine durchschnittliche Genesungsdauer von 14 Tagen an) zu bereinigen.

Abbildung 2 zeigt mit der hellblauen Kurve einen Verlauf der Epidemie unter Abwesenheit jeglicher öffentlicher Eingriffe. Die Zahl der Erkrankten steigt sehr schnell an, erreicht ihren Höhepunkte Anfang Mai 2020 und die Epidemie endet im Juli. Der Effekt der Bund-Länder-Beschlüsse vom 13. März 2020 (unter anderem Schulschließungen und Absage aller Sportveranstaltungen) und die daraufhin folgenden Maßnahmen werden durch den

Abbildung 3
**Zahl der Erkrankten in Deutschland im optimistischen Hubei-Szenario**

Anmerkung: Öffentliche Eingriffe werden in der Abbildung kurz ‚Kontaktsperre' genannt.

Quelle: Donsimoni, J. R., R. Glawion, B. Plachter und K. Wälde (2020), Projecting the Spread of COVID19 for Germany, https://www.macro.economics.uni-mainz.de/files/2020/03/Donsimoni-Glawion-Plachter-Wälde-2020-Projecting-spread-of-CoV-2-in-Germany-1.pdf (30. März 2020); nach Daten der Johns Hopkins University (USA).

Abbildung 4
**Zahl der insgesamt Erkrankten in Deutschland im optimistischen Hubei-Szenario**

Anmerkung: Öffentliche Eingriffe werden in der Abbildung kurz ‚Kontaktsperre' genannt.

Quelle: Donsimoni, J. R., R. Glawion, B. Plachter und K. Wälde (2020), Projecting the Spread of COVID19 for Germany, https://www.macro.economics.uni-mainz.de/files/2020/03/Donsimoni-Glawion-Plachter-Wälde-2020-Projecting-spread-of-CoV-2-in-Germany-1.pdf (30. März 2020); nach Daten der Johns Hopkins University (USA).

mittelblauen Verlauf dargestellt. Der Anstieg der Zahl der Erkrankten verlangsamt sich ab 20. März 2020, nimmt nach Ende der Maßnahmen wieder Fahrt auf und verläuft dann Anfang Juni über einen fast genauso hohen Gipfel. Das Ende der Epidemie verschiebt sich dann in den August. Bei einer Verlängerung der aktuellen Maßnahmen wird der Anstieg der Erkrankungen auf längere Zeit abgebremst, ist aber nach Ende der Maßnahmen wieder genauso schnell wie vorher. Der Gipfel ist später, wie auch das Ende der Epidemie – die nun für September zu erwarten wäre.

Grundsätzlich können wir festhalten: Die Politik der temporären Schließungen bzw. Einschränkung des Soziallebens ist wirksam. Die Reduzierung der sozialen Kontakte verringert die Ausbreitungsgeschwindigkeit von Infektionen und neuen Krankheitsfällen. Dafür wird aber auch das Ende der Epidemie hinausgezögert. Hinter diesen Berechnungen steht die Annahme, dass durch die Bund-Länder-Beschlüsse die Zahl der Kontakte in der Bevölkerung insgesamt um 50 % gefallen sind. Damit geht die Erkrankungsrate um 50 % zurück. Auch wenn es aktuell erste Anzeichen für die Wirksamkeit der Maßnahmen gibt (Hartl et al., 2020), muss noch ein bis zwei Wochen gewartet werden, ob diese Wirksamkeit von permanenter Natur ist.

## Wie lange dauert die Epidemie und wie stark wird diese?

Den Projektionen in Abbildung 2 liegt die Annahme zugrunde, dass langfristig 6 % der Einwohner Deutschlands an COVID-19 erkrankt und gemeldet sein werden. Damit ergibt sich die dramatisch hohe Zahl gleichzeitig Erkrankter aus Abbildung 2.

Im optimistischen Hubei-Szenario (vgl. Abbildung 3) gehen wir davon aus, dass parallel zu dem Anstieg der Erkrankten in Deutschland eine Immunisierung der Bevölkerung erfolgt. Diese findet über Infektionen statt, denen jedoch keine starken (oder sogar gar keine oder kaum wahrnehmbare) Symptome folgen. Dadurch wäre es möglich, dass am Ende dieser Epidemie tatsächlich nur 0,6 % der Bevölkerung erkrankt sind.

Im Falle einer unbeeinflussten Ausbreitung von SARS-CoV-2, erneut dargestellt durch die hellblaue Kurve, ergäbe sich auch in diesem optimistischen Szenario ein schneller starker Anstieg der Zahl der Erkrankten bis Ende April 2020. Danach würde die Zahl der Erkrankten wieder fallen und die Epidemie wäre Anfang Juni vorbei. Wenn wir von der aktuellen Situation mit Kontaktsperren ausgehen, dann befinden wir uns eher auf der mittelblauen Kurve. Wenn wir annehmen, dass die Kontaktsperre Mitte April aufgehoben wird, dann würde sich die Spitze der Zahl der Erkrankten auf Mitte Mai verschieben. Diese wäre mit um die 200.000 gleichzeitig Erkrankten auf einem Niveau, das unser Gesundheitssystem vor große Herausforderungen stellen würde. Wenn die Kontaktsperre um weitere sechs Wochen verlängert würde (die dunkelblaue Kurve), dann würde sich die Zahl der Erkrankten in der Spitze auf 120.000 verringern. Allerdings verlängert sich die Dauer der Epidemie dann auch bis Juli. In Abbildung 4 entspricht die Zahl der insgesamt Erkrankten den vom Robert Koch-Institut veröffentlichten Daten. Am Ende der Epidemie würde in allen Szenarien die Zahl der Erkrankten bei knapp einer halben Million liegen.

## Coronavirus in Deutschland: Szenarien und Hoffnungen

Wir befinden uns in Deutschland am Beginn einer Epidemie. Bei aller Unsicherheit bezüglich der Eigenschaften des Coronavirus-2 kann gesagt werden, dass die Epidemie noch bis in den Juni 2020 anhält. Kontaktsperren und ähnliche Politikmaßnahmen verlängern die Dauer der Epidemie. Im pessimistischen Szenario, was jedoch den Standardannahmen der Epidemiologie entspricht, dauert die Epidemie bis in den August hinein. Die Zahl der Erkrankten würde in diesem pessimistischen Szenario in die Millionen gehen.

Was bleibt zu hoffen? Wir haben vor wenigen Tagen gesehen (Hartl et al., 2020), dass die Bund-Länder-Maßnahmen vom 13. März 2020 die Zuwachsraten der Zahl der Erkrankten fast halbiert haben. Falls sich dieser positive Trend fortsetzt, könnten sich die Szenarien verbessern. Weiter bleibt zu hoffen, dass die Immunisierung in der Bevölkerung schneller voranschreitet als in „normalen“ Grippeepidemien. Auch dann könnte die Epidemie schneller zu Ende gehen. Oder weitere, noch strengere staatliche Gesundheitsmaßnahmen führen zu einem noch stärkeren Rückgang der Zuwachsraten. Die ökonomischen Kosten weiterer Eingriffe ins öffentliche Leben wären jedoch beachtlich und bereits aktuelle Schätzungen (Dorn et al., 2020) sind erschreckend. Es wäre eine Überlegung wert, Kontaktsperren auf die Bevölkerungsgruppen zu beschränken, die am ehesten einen starken Verlauf einer COVID-19-Erkrankung zu befürchten haben. Dies müsste von deutschlandweiten Unterstützungen (unter anderem Einkaufsservice und persönliche kontaktfreie Begegnungen) für diese Gruppen begleitet werden.

**Title:** ***Projecting the Spread of COVID-19 for Germany***

**JEL Classification:** *I18, E17, C63*
